# Identification of novel Cyclooxygenase-2-dependent genes in *Helicobacter pylori *infection *in vivo*

**DOI:** 10.1186/1476-4598-8-22

**Published:** 2009-03-24

**Authors:** Anna K Walduck, Matthias Weber, Christian Wunder, Stefan Juettner, Manfred Stolte, Michael Vieth, Bertram Wiedenmann, Thomas F Meyer, Michael Naumann, Michael Hoecker

**Affiliations:** 1 Department of Molecular Biology, Max Planck Institute for Infection Biology, Schumanstrasse 21/22 10117 Berlin, Germany; 2 Department of Microbiology and Immunology, University of Melbourne, Parkville, VIC, 3010 Australia; 3 Department of Hepatology, Gastroenterology, Charité Campus Virchow-Klinikum, Augustenburger Platz 1, 13353 Berlin, Germany; 4 Institute of Pathology, Klinikum Bayreuth, Preuschwitzer Strasse 101, 95445 Bayreuth, Germany; 5 Institute for Experimental and Internal Medicine, Faculty of Medicine, Otto-von-Guericke University, Leipziger Strasse 44, 39120 Magdeburg, Germany

## Abstract

**Background:**

*Helicobacter pylori *is a crucial determining factor in the pathogenesis of benign and neoplastic gastric diseases. Cyclooxygenase-2 (Cox-2) is the inducible key enzyme of arachidonic acid metabolism and is a central mediator in inflammation and cancer. Expression of the *Cox-2 *gene is up-regulated in the gastric mucosa during *H. pylori *infection but the pathobiological consequences of this enhanced Cox-2 expression are not yet characterized. The aim of this study was to identify novel genes down-stream of Cox-2 in an *in vivo *model, thereby identifying potential targets for the study of the role of Cox- 2 in *H. pylori *pathogenesis and the initiation of pre- cancerous changes.

**Results:**

Gene expression profiles in the gastric mucosa of mice treated with a specific Cox-2 inhibitor (NS398) or vehicle were analysed at different time points (6, 13 and 19 wk) after *H. pylori *infection. *H. pylori *infection affected the expression of 385 genes over the experimental period, including regulators of gastric physiology, proliferation, apoptosis and mucosal defence. Under conditions of Cox-2 inhibition, 160 target genes were regulated as a result of *H. pylori *infection. The Cox-2 dependent subset included those influencing gastric physiology (*Gastrin, Galr1*), epithelial barrier function (*Tjp1, connexin45, Aqp5*), inflammation (*Icam1*), apoptosis (*Clu*) and proliferation (*Gdf3, Igf2*). Treatment with NS398 alone caused differential expression of 140 genes, 97 of which were unique, indicating that these genes are regulated under conditions of basal Cox-2 expression.

**Conclusion:**

This study has identified a panel of novel Cox-2 dependent genes influenced under both normal and the inflammatory conditions induced by *H. pylori *infection. These data provide important new links between Cox-2 and inflammatory processes, epithelial repair and integrity.

## Background

*Helicobacter pylori *infection is associated with a variety of gastric disorders including chronic gastritis, peptic ulcer disease, mucosa associated lymphatic tissue (MALT) lymphoma, and gastric adenocarcinoma [1,2]. The pathogenicity of the bacterium is determined by epidemiological influences as well as bacterial and host factors [1,3]. Bacterial colonization of the gastric mucosa leads to development of a chronic inflammatory infiltrate, which is accompanied by enhanced release of inflammatory mediators, growth factors and reactive oxygen metabolites [2,4].

The inducible Cox-2 enzyme and its constitutively expressed isoform Cox-1 are the key regulators of human prostaglandin metabolism [5-7]. The end products of their enzymatic activity comprise a panel of prostaglandins and thromboxanes, which have been identified as critical regulators of fundamental physiological and pathological processes including platelet aggregation, parturition, T-cell development, inflammation and cancer [5-7]. Cox-2 enzymatic activity is largely regulated through *de novo *synthesis of Cox-2 protein [5,6].

In the stomach, enhanced *Cox-2 *expression has been found during *H. pylori*-triggered gastritis as well as in mucosal stress lesions, gastroduodenal ulcers and after ischemia/reperfusion damage [8-10]. Cox-2 and its related prostanoids also appear to contribute to the pathogenesis of gastric cancer. Gastric adenocarcinoma and premalignant mucosal lesions frequently over-express the *Cox-2 *gene [11-14], and elevated intratumoral Cox-2 levels seem to be associated with deeper tumor invasion [15] and an increased frequency of lymphatic metastasis [16]. In addition, Cox-2 inhibitors have been demonstrated to potently suppress proliferation of human gastric cancer cells *in vitro *[1,17,18] as well as experimental gastric adenocarcinomas in nude mice [17]. Recently however, a number of reports have challenged the notion that this anti- tumour activity is due to inhibition of Cox-2 itself [19]. Individuals taking Cox-inhibitors have been reported to display a reduced risk for development of gastric carcinoma [20], however the reported cardiovascular side effects associated with chronic coxib administration mean that clinical use of Cox- inhibitors for anti-carcinogenic treatment is controversial (Reviewed in[21]).

Expression of the *Cox-2 *gene therefore appears to be an important step in the pathogenesis of benign and malignant gastric diseases and therefore, clarification not only of its contribution to *H. pylori*-dependent pathogenesis, but also the downstream effects of Cox inhibiting drugs is of special clinical significance.

We have previously demonstrated that *H. pylori *can directly influence expression of *Cox-2 *in gastric epithelial cells through transcriptional mechanisms, and identified MAPK-ERK-dependent activation of a proximal *cis*-regulatory CRE-Ebox element as a key step in the *H. pylori*-response of the *Cox-2 *gene [22]. While these results further confirmed the pathophysiological link between the bacterium and *Cox-2*, molecular effectors located downstream of Cox-2 during gastric *H. pylori *infection remained unidentified.

Here we analysed gene expression in the gastric epithelium of mice treated with the Cox-2 specific inhibitor NS398, at different time points after *H. pylori *infection using DNA microarrays and were able to define gene expression profiles regulated by *H. pylori *through Cox-2-dependent and independent mechanisms.

## Results

### Determination of Cox-2 inhibitor concentration

To determine the appropriate concentration of inhibitor in our *H. pylori *infection model, PGE2 levels were measured in the gastric mucosa after *H. pylori *infection in the presence or absence of Cox-2 inhibition with NS398. Infected mice showed a 50% increase in PGE2 level in the gastric mucosa. Treatment of infected mice with NS398 (10 mg/kg) led to a reduction in the PGE2 such that it did not differ from the control group (data not shown). We therefore concluded that a dose of 10 mg/kg was sufficient to suppress Cox 2 activity in the presence of a *H. pylori *infection.

### Long term administration of the specific Cox-2 inhibitor NS398 does not significantly affect bacterial colonization or inflammatory scores

**Figure 1:**
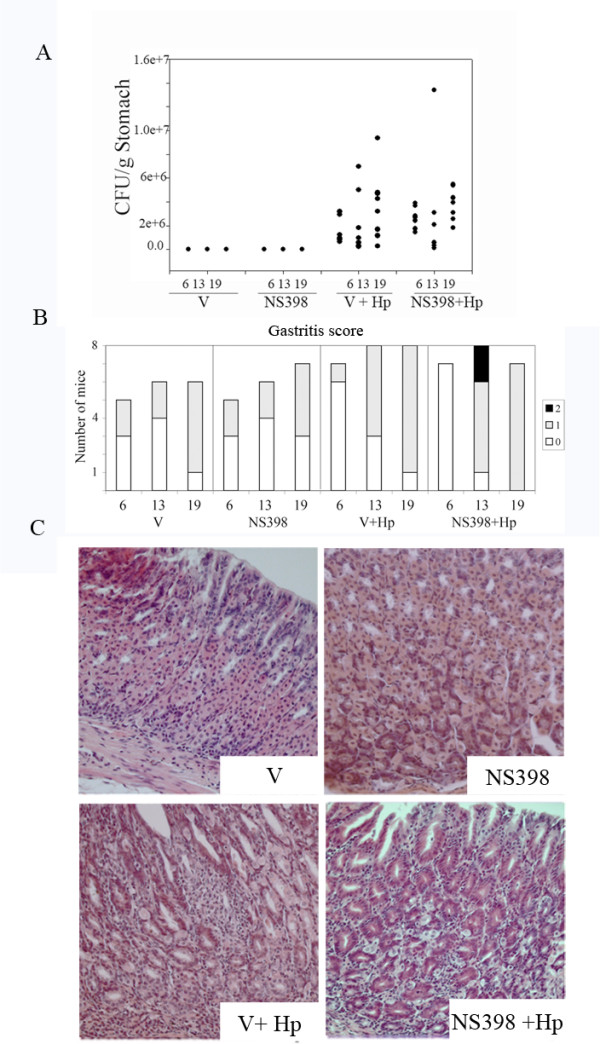
**Administration of the specific Cox-2 inhibitor NS398 does not significantly affect bacterial colonization or inflammatory scores**. **A**. Treatment with NS398 does not influence *H. pylori *colonization in C57 BL/6 mice. CFU retrieved from the stomachs of individual mice at 6, 13 and 19 weeks post infection (filled circles). **B**. Distribution of pathology scores in infected and control mice. Pathology was scored according to the Sydney system as follows: no inflammation (0), low grade, non -active gastritis and low grade mild-active gastritis (1), middle grade mild active gastritis (2). **C**. Hematoxylin and Eosin stained paraffin sections from 13 weeks post infection, representative images from non-infected mice treated with vehicle only (V), non infected mice treated with NS398 (NS398), infected mice (V+*Hp*), and infected mice treated with NS398 (NS398+*Hp*). By week 13 (17 weeks post-infection), moderate active gastritis was observed in both the V+*Hp *and NS398+*Hp *groups. Original magnification 20×, scale bar = 50 μm.

All mice in the infected groups were colonized with *H. pylori*, as determined by quantitative culture. The bacterial load increased only slightly in the period between 6 and 19 weeks (Figure 1A). Administration of NS398 did not appear to have a significant effect on bacterial colonization. Infection with *H. pylori *caused a low to middle grade gastritis in infected mice that tended to increase in severity over time, but did not lead to ulcer formation or evidence of metaplasia (see Figure 1B, and Figure 1C for comparisons of mice in each group at week 13). These observations are in accordance with reports from other studies where mice were infected for similar periods of time [23,24]. Histological analysis showed administration of vehicle and NS398 alone induces a low-grade gastritis over time (Figure 1B).

**Figure 2:**
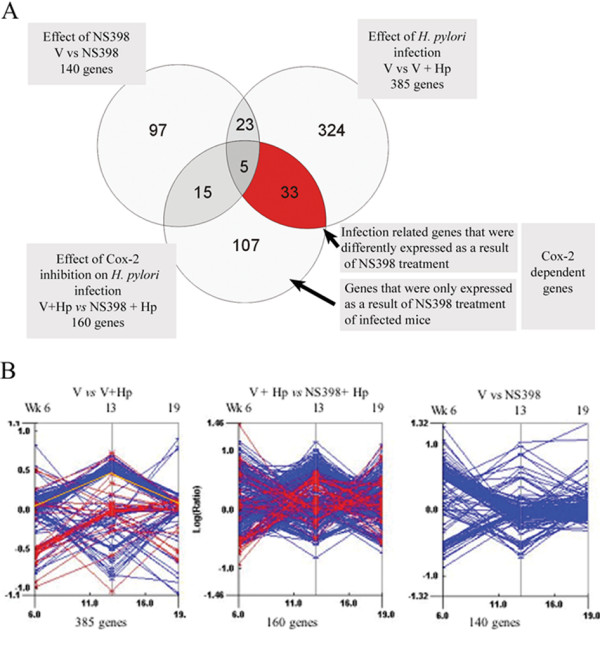
**Global gene expression in the gastric mucosa of mice infected with *H. pylori***. **A**. Venn diagram illustrating the breakdown of differentially expressed genes (more than 3 fold up or down) over the period of the study. The number of genes passing the cut-off criteria is indicated for each experimental comparison: Infected versus non-infected mice (V *vs *V+*Hp*), infected mice versus NS398 treated and infected mice (V+*Hp vs *NS398+*Hp*), and non-infected mice versus NS398 treated (V *vs *NS398). Numbers in parentheses represent the number total genes differently expressed in the three experimental comparisons. **B**. Trend plots representing global gene expression measured in the 3 experimental comparisons. Each point on the y-axes represents the average expression ratio for genes passing the cut-off criteria at weeks 6, 13 and 19 after treatment starts, i.e. one line represents one gene. The 33 infection-related genes that were differently expressed as a result of NS398 treatment are highlighted in red for all comparisons. Cox-2 (Ptgs2) is indicated by the yellow line.

RNA from animals with similar scores and colonization levels (3 mice per group) were pooled and used to perform the three experimental comparisons: non-infected versus infected (V *vs *V+*Hp*), infected versus NS398 treated and infected (V+*Hp vs *NS398+*Hp*), and non-infected versus non-infected and NS398 treated (V *vs *NS398) (Figure 2A). The experiment was designed to enable us to determine gene expression profiles in the stomachs of mice receiving vehicle alone or NS398 in vehicle, and to isolate these from the effects of *H. pylori *infection.

### Global gene expression in the gastric mucosa of *H. pylori *infected mice

The RNA used in this study was extracted from the gastric *mucosa *only, and histological analyses of stomachs prepared in this way have confirmed that muscle or other connective tissue underlying the mucosa were not included in our preparations (not shown). The changes in gene expression seen in this study are therefore highly likely to reflect a transcriptional response restricted to that of gastric epithelial cells and the developing lymphocytic and granulocytic infiltrates which characterise chronic *H. pylori *infection. Cut-offs for significant changes in gene expression were set at p < 0.05 and three fold change [25].

In *H. pylori *infected mice, 385 genes passed the cut- off criteria at at least one time during the study (Figure 2A). *H. pylori *infected mice that were treated with NS398 had 160 differentially expressed genes. In mice treated with NS398 alone, 140 genes were differentially expressed (Figure 2A). Using a subtractive approach, genes were divided into subgroups reflecting the major experimental effects: *H. pylori *infection, Cox-2 suppression, and Cox-2 suppression during infection.

Infection with *H. pylori *induced a complex pattern of global gene expression over the period of the experiment (Figure 2B). Treatment of infected mice with NS398 resulted in a distinct gene expression signature, which was additional to the effect of infection. Thirty-three of the genes differently expressed in infected mice was a result of NS398 treatment (red highlighted genes), in addition a further 107 genes were only expressed in NS398 treated and infected mice. In this manner a subgroup of genes defined Cox-2 dependent genes was established. We subdivided these genes into subgroups to facilitate further analysis based on the Gene Ontology categories using the DAVID tool for gene annotation at the NCBI . Table 1 contains selected Cox-2 dependent genes (a complete list may be found in Additional File 1, Table S2). Most genes showed a fluctuating expression pattern indicating that control mechanisms and the cumulative effects of both infection and Cox-2 suppression play a role in gene expression over time.

**Table 1:** Experimental design

**Week**		**-4 & -3**	**0**	**6**	**13**	**19**
	**Mice (n)**					

Vehicle only (V)	17	Mock infection	Treatment begin	5	6	6

NS398 (10 mg/kg) (NS398)	17	Mock infection	Treatment begin	5	6	6

Vehicle plus *H. pylori *(V+ *Hp*)	24	Infect SS1	Treatment begin	8	8	8

NS398 (10 mg/kg) plus *H. pylori* (NS398+*Hp*)	24	Infect SS1	Treatment begin	8	8	8

After week 0 mice received daily sc. injections of NS398 or vehicle alone as indicated. Mice were killed at 6. 13 and 19 weeks after the start of treatment and *H. pylori *colonization (cfu), then pathology scores were determined and total RNA was prepared.

### Effect of NS398 on gastric gene expression profiles

Previous studies on the Cox-2 inhibitor NS398 in a variety of *in vitro *and *in vivo *models have demonstrated it is a specific inhibitor of Cox-2 activity with little or no influence on the closely related, constitutively expressed *Cox-1 *[26,27]. In our study, long-term administration appeared to have effects on gene expression that may have been resolved or compensated for after several months, as a large proportion of the genes which were up-regulated after week 6 of administration were not differently expressed after week 13 (Figure 2B and Table 1). Only 33 of the genes that were influenced by NS398 were also regulated in response to infection. A complete list of the genes regulated as a result of NS398 treatment is shown (Additional File 1, Table S3.

**Figure 3:**
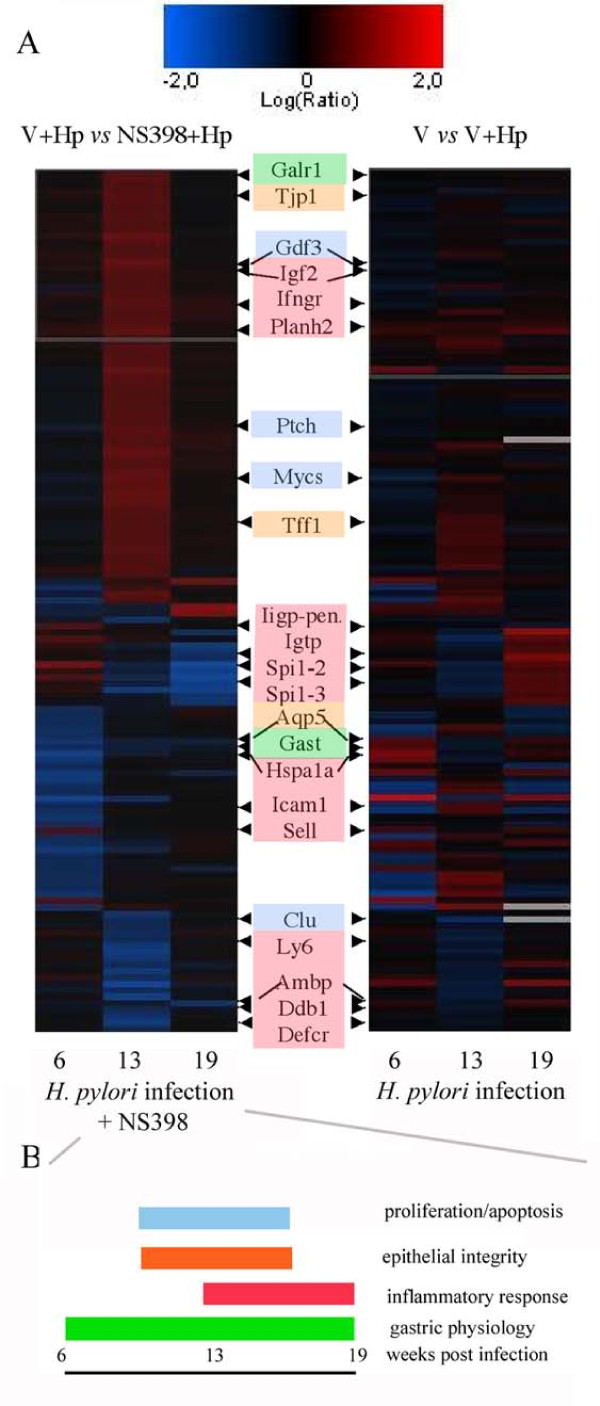
**Expression pattern of Cox-2 dependent genes**. **A**. Two-dimensional self-ordering matrix (SOM) cluster showing relative expression level of all Cox-2 dependent genes identified in this study (p > 0.05). Up- or down-regulations are indicated by red, or blue shading respectively. Black shading indicates similar gene expression in both samples. The right panel shows the cluster derived from the gene expression pattern in NS398 treated mice (V+*Hp *vs NS398+*Hp*). The left panel shows the expression of the same genes in infected mice (unclustered). Gene functions and literature references relevant to *H. pylori *infection are shown in Table 3. Genes fitting the most prominent functional categories are highlighted with colour: proliferation/apoptosis (pale blue), epithelial integrity (orange), inflammatory response (red) and gastric physiology (green) **B**. Diagram summarizing the overall physiological effect of Cox-2 dependent genes over the period of the experiment, gene categories are coloured as in A.

To complement the 'subtractive' method of classifying genes, data were clustered using an unsupervised approach. A self-ordering matrix (SOM) was created using all Cox-2 dependent genes, Figure 3 shows an SOM plot of the Cox2-dependent genes identified in the study (left panel), the right panel shows a matrix of the same genes to indicate the effect of *H. pylori *infection alone on the same genes (See also Table 2). Broadly, the regulated genes fell into the functional categories of being related to epithelial barrier function, proliferation and maintenance or inflammation (summarized in Figure 3B).

**Table 2:** Fold change in gene expression of selected genes regulated as a result of infection with *H. pylori *and/or treatment with the specific Cox-2 inhibitor NS398

***SYMBOL***	***GENE NAME***	***GENE ONTOLOGY***	**V *vs *V + *Hp***			**V+*Hp vs *NS398+*Hp***			**V *vs *Ns398**		
			
			6	13	19	6	13	19	6	13	19
Adn	adipsin	chymotrypsin, activity. complement activation.	3.06	1.9	-1.32	-2.4	1.93	-1.07	-1.53	-2.56	-1.97

Akt3	thymoma viral proto-oncogene 3	protein amino acid phosphorylation	-1.52	6.68	-1.02	-1.09	1.28	1.33	-1.44	-1.03	-1.06

AV148957	Connexin 45	water transport	1.39	-3.25	-1.04	1.29	-1.11	1.39	1	1.09	-1.01

Ccl5	chemokine (C-C motif) ligand 5	chemokine activity. inflammatory response	1.23	-1.13	3.01	1.08	-1.13	-1.54	-1.21	1.48	1.08

Crpd. Dmbt1	deleted in malignant brain tumors 1/crp ductin, muclin	scavenger receptor activity. tumor suppressor	4.15	-3.15	7.74	-2.64	-2.09	-2.15	1.78	1.28	-1

Gast	gastrin	hormone activity	6.37	-1.21	1.8	-8.41	-1.87	-1.01	1	-2.35	1.45

Hsp70-3	heat shock protein 1A	chaperone activity	5.3	-1.86	1.31	-5.41	1.13	-1.15	3.23	-1.35	1.11

Icsbp1	interferon consensus seq. binding protein 1	immune response; transcription regulation	1	-1.31	3.36	1.19	1.23	-1.39	1	2.04	-1.37

Ifi47	interferon gamma inducible protein	ATP binding activity	1.3	-1.29	6.03	1.2	-1.13	-1.97	-1.85	-1.29	1.45

Ly75	lymphocyte antigen 75	defense response	1.23	-2.25	3.37	1.36	-1.15	1	1	1	1.39

Mup5	major urinary protein 5	pheromone binding activity; transport; transporter activity	4.94	-1.08	3	-6.56	-1.23	-2.5	7.56	-1.5	1.31

Ptgs1	prostaglandin-endoperoxide synthase 1	prostaglandin biosynthesis	-1.36	-1.03	-1.02	1.16	1.03	1	1.26	1.47	1.12

Ptgs2	prostaglandin-endoperoxide synthase 2	prostaglandin biosynthesis	1.27	4.34	1.47	-1.02	1.17	1	-1.55	-1.24	-1.47

Odc	Ornithine decarboxlyase	carboxy- lyase activity	1.05	-1.69	-1.19	-1.34	-3.12	-1.11	1.94	-1.4	1.23

Slc7a11. cd98 light	solute carrier family 7 member 11. CD98 light	cationic amino acid transporter	1.37	3.42	1.19	1.09	2.24	1.33	-1.7	-1.64	-1.07

Tff1	trefoil factor 1	response to wounding	-2.94	5.57	-3.06	-2.28	9.16	-1.56	-1.37	-6.56	2.84

Tgtp	T-cell specific GTPase	GTP binding activity	1.26	-1.24	8.29	1.55	-1.17	-2.72	-1.37	1.31	1.43

The Gene Ontology (GO) classification indicates gene function. A complete list of the expression level of all genes in the study may be found in Additional File 1, Tables S1-3.

### Confirmation of expression of selected Cox-2 dependent genes

The mean change in expression level of *Cox1 (Ptgs1) *and *Cox2 (Ptgs2)*, and a selection of Cox2-dependent genes involved in inflammation (intracellular adhesion molecule 1, *Icam1*; Transforming growth factor b1,*Tgfb1*), gastric function (Gastrin, *Gast*), barrier function (Aquaporin 5, *Aqp5; *Tight junction protein 1, *Tjp1*) and proliferation/carcinogenesis (Ornithine decarboxylase, *Odc1*) was determined for individual mice at all time points by real-time PCR (Additional File 1, Table S4). In 94% of cases the change in expression could be confirmed (up- or down- regulated over the three-fold cut off criteria).

**Figure 4:**
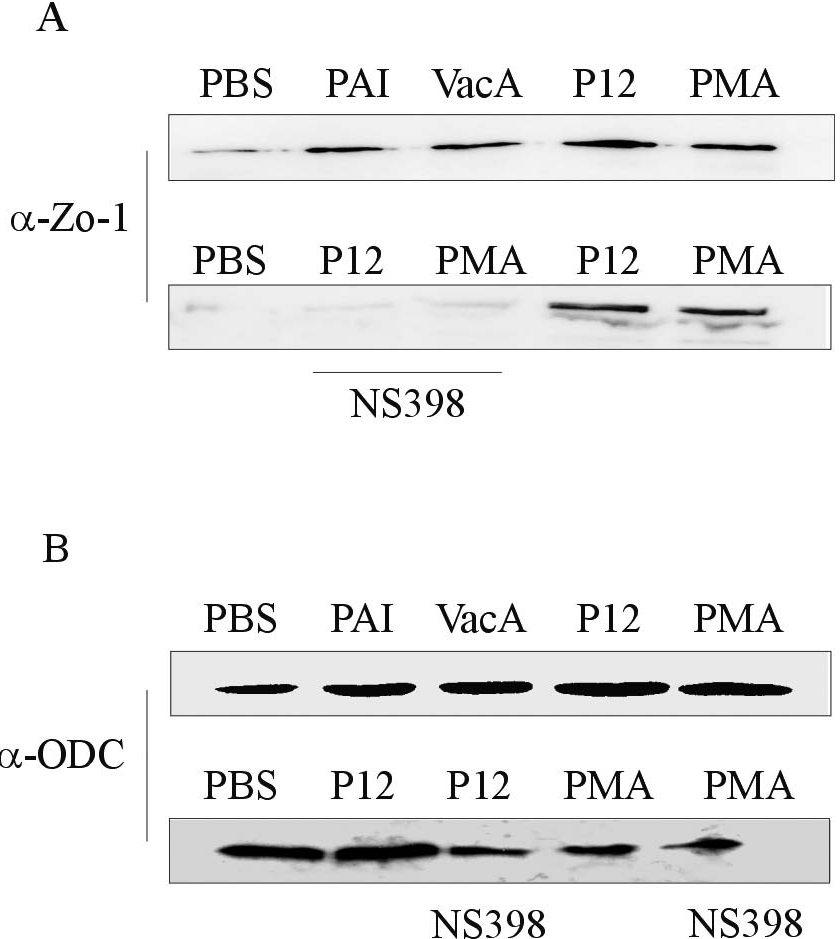
**Cox-2 dependent expression of ZO1 and Ornithine decarboxylase in MKN28 cells dependent in *H. pylori *infection**. **A**. Expression of zona occludens-1 (ZO1) was determined by western blot at 6 h post infection in MKN28 cells with either *H. pylori *wild type P12 or the isogenic mutants missing the entire cag PAI (PAI), or VacA (VacA). ZO1 was up regulated after infection, independent of CagA PAI or VacA (upper), whereas the expression was decreased in cells treated with NS398 (lower). **B**. Expression of Ornithine decarboxylase protein (ODC) was not affected by *H. pylori *infection, but was decreased in the presence of NS398.

In NS398 treated mice, the expression of *Tjp1 *dramatically increased in the early stages of the study compared to infected mice (Figure 3). The mouse EST homologous to connexin 45 (AV148957, Gja7) was also decreased in infected mice, regardless of NS398 treatment, as was a gastric aquaporin Aqp5 (Figure 3). This suggested that Cox-2 inhibition has an effect on the *H. pylori *mediated influence of gastric barrier function, therefore we investigated this further in an *in vitro *model. Western blot analysis showed that infection with *H. pylori *also led to increases in Zona occludens 1(ZO1, Human homologue to Tjp1) protein expression *in vitro *in MKN28 gastric epithelial cells, and that this increase was inhibited in the presence of NS398 (Figure 4A). Interestingly, this effect was independent of the presence of an intact type IV secretion system, or the presence of the cytotoxin VacA, as *H. pylori *deletion mutants also induced ZO1 expression (Figure 4B). Expression of ODC was decreased in MKN28 cells treated with NS398 (Figure 4C), and was also independent of the presence of a functional type IV section apparatus or VacA.

## Discussion

The aim of this study was to enhance our knowledge about the role of cyclooxygenase-2 in *H. pylori*-triggered mucosal inflammation by identifying new downstream molecular effectors. The *in vivo *approach taken here has also provided insight at the transcriptome level, into the complex changes that occur as a result of the chronic inflammatory response to *H. pylori *infection and to Cox-2 inhibition.

Both *H. pylori *infection, and NS398 treatment elicited unique transcriptional signatures in the gastric mucosa of mice, despite the similarity in pathology scores and colonization density between the different groups (Figure 1). This is of interest because in another report employing a model of mucosa-associated lymphoid tissue lymphoma (MALT lymphoma), transcriptional profiles in BALB/c mice infected with *H. heilmannii *were determined after 12 to 24 months, and the major changes in gene regulation occurred in the earlier stages of the disease (<12 months, mild to moderate pathology) [28]. After this time, clustering of the 300 most differentially expressed genes allowed segregation of infected mice into groups corresponding almost exactly with their pathological characterization. In view of our data, we would conclude that during the early development phase of chronic active gastritis, standard pathology analysis is not able to detect subtle, but important changes in the mucosa.

NS398 specifically inhibits the activity of the Cox-2 protein, and alterations in *Cox-2 *gene expression might be expected when infected mice were treated with a specific Cox-2 inhibitor, either as a result of a possible feedback mechanism involving PGE_2 _[29], or as a compensatory mechanism to overcome the enzymatic inhibition. No significant change in *Cox-2 *expression was observed *in vivo *however, supporting the notion that the expression of the *Cox-2 *gene in the stomach is controlled by a variety of factors. Cox-2 is expressed by both inflammatory and gastric epithelial cells [22] and its expression may be controlled by different mechanisms in different cells types.

We were able to identify a subset of genes that were differentially expressed as a result of Cox-2 suppression, highlighting the scope of influence of Cox-inhibitors in gastric inflammation. Cox-2 dependent genes fell into numerous functional categories, chiefly those involved in gastric physiology (acid secretion, motility), epithelial repair and proliferation, and inflammatory mediators.

Gastrin (*Gast*) is an important mediator in the stomach [30-34] and expression in the mucosa was strongly influenced, not only by infection with *H. pylori*, but also by suppression of Cox-2 activity (Figure 3, Additional File 1, Table S4). In addition to its role in regulating gastric acid secretion, gastrin has trophic effects and regulates proliferation and repair in the mucosa. Indeed INS-GAS transgenic mice which suffer from hypergastrinemia develop carcinoma after infection with *H. pylori *[35]. The development of carcinoma is however restricted to males in this model [35]. Other workers have also observed that Cox-2 inhibition influenced gastrin expression in an *in vitro *colorectal cancer model [34] and also in *H. pylori *positive gastric cancer patients [36].

Whereas expression of the apoptosis mediating growth differentiation factor 3 (*Gdf3*) and c-Myc (*Mycs*) genes peaked at week 13, the apoptosis inhibiting gene clusterin (*Clu*) strongly decreased at this time point. Taken together, the gene expression pattern is suggestive of a shift in the rate of proliferation/apoptosis of the epithelium after 13 weeks of infection as a result of NS398 treatment. Longer-term studies would be required to determine whether this effect continues or recurs. A number of genes which have been previously observed to be over-expressed in tumours (*Mycs, Clu*)[37], tumor suppressors (Patched, *Ptch*), or otherwise involved in metastasis or DNA repair: bikunin (*Ambp*)[38], ornithine decarboxylase gene (*Odc*), Trefoil factor 1 (*Tff1*)[39], insulin like growth factor (*Igf2*) [40] and DNA repair protein 1 (*Ddb1*), were also differentially expressed in NS398 treated infected mice (Figure 3). The expression pattern was complex however, and some mediators tended to be enhanced by Cox-2 suppression, while the majority were down-regulated (Figure 3). This probably reflects a regulatory role for Cox-2 as part of a network of control mechanisms for epithelial maintenance. The *Odc *gene, for example, encodes a key regulatory enzyme in the production of polyamines which are essential for cell proliferation [41] and has been shown to play a role alongside Cox-2 in the development of atrophic gastritis [42,43]. It has been proposed that Cox-2 inhibitors may inhibit Odc, and in this way be responsible for the observed anti-proliferative effects of Cox-2 inhibitors [44]. Our observation of decreases in Odc expression as a result of NS398 treated *H. pylori *infection in both *in vivo *and *in vitro *studies (Table 1 and Figure 4C) is in keeping with this notion.

*Helicobacter *infection is strongly associated with the induction of a strong Th1-type inflammatory response, with high levels of Ifnγ, which induces expression of other inflammatory mediators such as iNOS and *Cox-2*, and also circulating growth factors such as gastrin [45]. Supporting the notion that Ifnγ plays the key role in attracting and activating lymphocytes [46,47], we observed that expression of T-cell surface markers *Icam1 *[48,49] and *Cd86 *and ligands (*Sell*) [50] peaked at week 13 in infected mice. The interferon dependent GTPases (*Igtp*, *Iigp-pending*) regulate the anti-microbial activities of Ifnγ in a STAT1 dependent manner [51,52], and their expression was dramatically reduced in NS398 treated mice by the end of the study. Overall, inhibition of Cox-2 activity led to a reduced expression of inflammatory mediators between weeks 13 and 19 (Figure 3); interestingly this change was not reflected in the pathology scores. Cox-2 has been shown to modulate the Th1/Th2 balance in inflammatory responses and inhibition of Cox-2 using NS398 led to a polarization of the response of *in vitro *stimulated human PBMCs toward Th1[53]. The authors postulated that chronic expression of Cox-2 and production of PGE2 results in an inhibition of the effectiveness of the mucosal immune response by enhancing a state of tolerance. The gene expression pattern we observed here is indeed consistent with an effect of Cox-2 inhibition on the inflammatory response (See Figure 3 and Table 1) although the changes in expression of classical Th1/Th2 mediators such as IL-12, I-10 and IL-4 did not differ significantly in our study.

Infection with *H. pylori *has been reported to damage epithelial integrity and several potential mechanisms for this have been reported (reviewed in [54]). CagA, a major *H. pylori *pathogenicity factor, is translocated into epithelial cells via the type IV secretion apparatus [55,56]. Studies in a canine kidney cell model (MDCK) showed that CagA associates with the tight junction adaptor protein zona occludens 1 (zo-1, mouse homologue -tight junction protein 1, Tjp1) and the junctional adhesion molecule (Jcam or Jam), leading to a long term disruption of epithelial barrier function *in vitro *[57]. Whilst we observed increased expression of *Tjp1 *at the transcriptional level in NS398 treated infected mice, *Jcam *expression was not affected. In addition, an EST with homology to connexin 45 (AV148957, *Gja7*) and *Aqp5 *were influenced by *H. pylori *infection regardless of NS398 treatment (Figure 3). As both connexin 45 and Aqp5 are know to play a role in intercellular transport of water and small molecules, and there is experimental evidence that connexin 45 interacts directly with Tjp1 [58,59], it would appear that Cox-2 also has a role in the maintenance of water balance in the gastric epithelium. This idea is supported by our *in vitro *observations of a cox-2 dependent increase in Zo-1 protein expression in MKN28 cells. In contrast to the reports by Amieva et al. [57] this effect was not related to the CagA status of *H. pylori *(Figure 4). Barrier function effects in the mouse model are in any case unlikely to be due to the actions of CagA, as although we found that *H. pylori *SS1 expressed CagA protein, we were not able to detect translocation in either *in vitro *or *in vivo *experiments (data not shown). Therefore, it would appear that *H. pylori *infection has additional mechanisms to influence epithelial integrity. It is also of note that another *H. pylori *pathogenicity factor, vacuolating toxin (VacA) causes formation of fluid-filled vacuoles in epithelial cells and furthermore, this activity can be inhibited *in vitro *by NS398 treatment [60]. As the gastric aquaporin Aqp5 is expressed on the lateral and intercellular membranes in the gastric crypts [59], we speculate that this pore is influenced by changes to tight junction proteins, and that it plays a role in the development of oedema in the epithelium during infection.

A number of published reports have attempted to shed light on gene regulation in *H. pylori *infection using the microarray approach to study global gene expression in gastric epithelial cells *in vitro *[61-63] (reviewed in [64]), reporting a rapid up-regulation of inflammatory mediators and a variety of transcription factors to be the hallmarks of the expression pattern. In our study, none of the proliferation related genes (*c-Fos, b-Fos, c-Jun *and *cyclinD1 *(*Pcna*)) reported by Sepulveda *et al*., nor those reported by Cox *et al*. (amphiregulin, *Adam10*) [61] were differentially expressed in any group of mice. This probably reflects the differences between the *in vitro *and *in vivo *models of infection. The data reported here, and that of other reports [25,65] strongly suggest that cells of the immune system influence not only this effect but also epithelial integrity, limiting the conclusions that can be drawn from *in vitro *studies using cell lines in isolation.

## Conclusion

Some of the Cox-2 dependent genes from this study have been previously identified as being influenced by Cox-2 or Cox-2 inhibition (*Gast, Icam1, Odc*), but the remainder are novel and point to new links between Cox-2 and inflammatory processes, epithelial repair and integrity. This study has provided insights into not only the effects of prostaglandin inhibitors on inflammation, but also enabled us to identify novel Cox-2 dependent processes such as a role in epithelial integrity and as a result further targets for its role in transformation. Further investigation of these targets will help us to explain the protective effects of Cox-2 inhibitors and NSAIDs in gastritis patients and aid the development of new treatment strategies.

## Methods

### *H. pylori *strains

*H. pylori *strain P12, ΔPAI and ΔVacA were cultured on GC Agar plates as previously described [66,67]. The mouse adapted *H. pylori *strain (SSI) (kind gift from A. Lee, University of Sydney, Australia) were cultured on GC Agar plates as previously described [68]. For infection, bacteria were scraped off plates and grown overnight in liquid culture in Brain Heart Infusion (BHI) broth [68].

### Detection of prostaglandin E2 in stomach after daily administration of NS398

After infection with a single oral dose of 1 × 10^9 ^of a mouse adapted strain of *H. pylori *(SS1) suspended in sterile PBS, female C57 BL/6 mice (n = 18) then received daily subcutaneous injections of either vehicle (1% Tween 80) or 10 mg/kg of NS398 (Merck) in 1% Tween 80 for 14 days. On day 14, mice were euthanized and the stomach was removed, opened, washed in ice cold PBS, and frozen in liquid nitrogen. The gastric mucosa was scraped off and pulverized in liquid nitrogen. The material was weighed, suspended in 0.1 M phosphate buffer, vortexed for 1 min, and then clarified by centrifugation at 10 000 g for 15 min at 4°C. The supernatant was purified using a Prostaglandin E2 Affinity Column (Cayman) according to the manufacturer's instructions. The amount of prostaglandin E2 was determined by enzyme immunoassay (PGE2 EIA kit; Cayman).

### Effect of administration of NS398 on inflammation in chronic *H. pylori *infection

For the long-term gene expression study, 48 female C57 BL/6 mice were infected with two oral doses (one week apart) of 1 × 10^9 ^*H. pylori *(SS1) and 100 μl 0.1 M bicarbonate. Control (non-infected) mice received bicarbonate alone. Groups of infected and non-infected mice received daily subcutaneous doses (100 μl) of 10 mg/kg NS398 dissolved in PBS, 1% Tween 80 for 6, 13 or 19 weeks. Control groups of mice received vehicle alone (PBS, 1% Tween 80) (Table 3).

**Table 3:** Function of selected Cox-2 dependent genes shown in Figure 3

**Gene**	**Function. Remarks**	**Reference**
Galr1	Receptor for galanin, pain perception, inhibits basal and gastrin-induced acid secretion	

Tjp1	Tight junction protein, epithelial integrity. *H. pylori *interaction	[57]

Gdf3	Regulator of cell growth and differentiation	

Igf2	Cell growth/control	[40]

Ifngr	Inflammation	[46]

Planh2. Serpinb2	Inhibits plasminogen activator. important role in cell matrix degradation. gastrin dependent	[32,30]

Ptch	Cell fate. tumor suppressor	

Mycs	Oncogene. mediates apoptosis	[37]

Tff1	Protects mucosa from insults. stabilizes mucus layer. epithelial healing. suppresses proliferation	[39]

Iigp- pend. & Igtp	Regulates anti-microbial activities of IFNγ in a STAT 1 dependent manner	

Spi1-1-3	Tissue scavenger of leukocyte elastase	

Aqp5	Water transport.	

Gast	Regulation of gastric acid secretion. Growth factor activity in gastric mucosa. Selective inhibition of Cox-2 reverses trophic activity.	[31,33,34]

Hspa1a	Heat shock protein, chaperone.	

Icam1	Mediates lymphocyte migration.	[48,49]

Sell. L-selectin	Mediates lymphocyte migration	[50]

Clu	Inhibits apoptosis, levels elevated in both mouse and human tumours	

Ly64, MUC13	In concert with TLR4, controls B cell recognition and signalling of lipopolysaccharide (LPS)	

Ambp, bikunin	Suppresses urokinase expression and tumour metastasis	[38]

Ddb1	DNA repair protein	

Defcr	Anti microbial peptide	[25]

Literature references are shown for genes previously reported or investigated in connection with *H. pylori *infection.

### Preparation of stomach tissue

Mice were killed and the stomach was removed and cut along the greater curvature into two tissue fragments encompassing antral and oxyntic mucosa, as well as part of the non-secretory epithelium. The stomach was cut longitudinally into 3 parts. One third of the tissue was frozen immediately in liquid N_2_. One third was weighed, homogenized and then serially diluted and plated out on Blood Agar plates (blood agar base No.2, 10% defibrinated horse blood, Vancomycin, Amphotericin B, Polymyxin B, Bacitracin, Nalidixic acid) to determine the number of colony forming units (cfu) of *H. pylori*. The remaining 1/3 of tissue was fixed in 4% buffered paraformaldehyde and processed for histological analysis. Tissue was embedded in paraffin and 2–4 μm sections were stained with hematoxylin and eosin, plus Warthin Starry stain and assessed by 2 independent pathologists (M.S & M.V) blinded to the experimental design. Pathology and colonization were scored according to the modified Sydney System [69].

### RNA isolation and quality control

RNA was isolated from 1/3 the gastric mucosa by scraping tissue over liquid nitrogen as previously described [25]. RNA from 3 mice with similar colonization levels were selected and pooled for microarray analysis.

### In situ Oligonucleotide Arrays

A custom oligonucleotide glass array of specific 60 mer oligonucleotides representing 8187 mouse genes was designed based on Unigene Clusters (Unigene build#148), and produced by Agilent Technologies (Palo Alto, USA)[25]. Oligonucleotide probes were synthesized and deposited by Agilent using *in situ *ink-jet printing technology as proposed by Blanchard *et al*. [70].

### Labeling and hybridization to arrays

RNA (5 μg) from each pool was amplified and labelled as described previously [25]. Pairs of target RNAs were mixed and hybridized to arrays to perform 3 experimental comparisons i.e. infected versus non infected, infected only versus NS398 treated and infected and non infected versus non infected and NS398 treated (Figure 2A). After data extraction and normalization [25] data from duplicate hybridizations were combined and expressed as log ratios or fold changes. Statistical and bioinformatic analysis was performed using the Rosetta Resolver™ software package (V 3.2 (build 3.2.0.2.40)), Rosetta Biosoftware, Kirkland, USA).

### Culture of gastric cell lines, and in vitro infection experiments

MKN28 cells (ATCC, Rockville, USA) were cultured in RPMI 1640 (Gibco BRL, Eggenstein, Germany) medium containing 10% foetal calf serum (FCS, Invitrogen, Carlsbad, Germany) using standard methods. *H. pylori *strains P12, P12-PAI and P12-VacA were added to epithelial cells at a multiplicity of infection (MOI) of 100. Western blots were performed as described previously [22]. Proteins were transferred to a PVDF membrane (Perkin Elmer, Jügesheim, Germany) and detected using specific antibodies (Cox-2, Cayman, MI, USA; Zo1; Odc and Aquaporin 5, Santa Cruz, CA, USA,). An HRP-labelled secondary goat anti-rabbit antibody was used (diluted 1:3000) and the Renaissance Western Blot system (ECL, Eschwege, Germany) according to the manufacturer's recommendations.

### Real-Time PCR assay

Total RNA was isolated from tissue as described above and subsequently used for cDNA synthesis. Real-Time quantitative RT-PCR analyses for Cox-2 sense 5'-AGA AGG AAA TGG CTG CAG AA-3, antisense 5'AGG TGC TCG GCT TCC AG TAT); Aqp5 sense 5'GGC CCT CTT AAT AGG CAA CC-3, antisense 5'TTG CCT GGT GTT GTG TTG TT-3; Gast sense 5' ACC AAT GAG GAC CTG GAA CA-3, antisense 5'CAT CCA TCC GTA TGC TTC CT-3; Tjp1 sense 5'-TCC ACC TCT GTC CAG CTC TT-3', antisense 5'-CAC CGG AGT GAT GGT TTT CT-3'; Icam1 sense, 5'-TTC ACA CTG AAT GCC AGC TC-3' antisense 5'-GCC ACA GTT CTC AAA GCA CA-3'; Odc sense 5'-TTG CCA CTG ATG ATT CCA AA-3', antisense 5'-AGC CAC CAC CAA TAT CAA GC-3' and TGFβ sense 5'-ACC TTC TGA TCC ATC GGT TG-3' TGFβ antisense 5'-TTC CTG TTG GCT GAG TTG TG-3' cDNA were performed using the ABM PRISM 7700 Sequence Detection System instrument and software (PE Applied Biosystems, Inc., Foster City, CA) and SYBR Green PCR Master mix (Applied Biosystems) with thermocycler conditions recommended by the manufacturer. PCRs were performed in duplicate in a total volume of 30 μl containing 10 μM primers. Data were normalized to *HPRT *(hypoxanthine phosphoribosyl transferase) expression to perform relative quantifications (Primers: sense 5'-GTT GGA TAC AGG CCA GCA TTT GT-3', antisense 5'-CAC AGG ACT ACT AGA ACA CCT GC-3'). Relative gene expression was calculated using the method described by Pfaffl [71] and expressed as fold change.

## Competing interests

AW has served as a consultant for Agilent Technologies (the MPI for Infection Biology was a reference laboratory for Agilent Technologies). The authors declare that they have no other competing interests.

## Authors' contributions

MN and MH conceived the studies, oversaw the experimental work and helped draft the manuscript. AW, MW, CW and SJ performed the experimental work and participated in data analysis. AW analyzed the microarray data and wrote the manuscript. MS and MV performed the pathological scoring. BW and TFM contributed to the design of the studies. All authors read and approved the final manuscript.

## Supplementary Material

Additional file 1 **Tables S1, S2, S3 and S4**. **Table S1 **– Fold Change in gene expression of all genes in infected mice (V+*Hp*) relative to non-infected, vehicle only treated mice (V) ie. A fold change of 3 means that the gene was 3 times more strongly expressed in infected mice (V+*Hp*). **Table S2 **– Fold Change in gene expression of all genes in infected, NS398 treated (NS398+*Hp*) relative to infected mice (V+*Hp*). **Table S3 **– Fold Change in gene expression of all genes in in non infected, NS398 (NS398) relative to non infected, vehicle only treated mice (V) mice. **Table S4 **– Confirmation of gene expression using Real time PCR. The expression level of selected genes was confirmed by performing semi quantitative real time PCR on cDNA from individual mice. Fold changes are the man fold change from n = 3 mice. In all but 4 instances, the microarray result was confirmed (cut off ± 3 fold change) and even in these cases, the gene expression had the same tendency (up or down regulated) (see italicised data points). Click here for file
